# Molecular and Functional Profiling of Gαi as an Intracellular pH Sensor

**DOI:** 10.21203/rs.3.rs-4203924/v1

**Published:** 2024-04-30

**Authors:** Ajit Prakash, Zijian Li, Venkata R. Chirasani, Juhi A. Rasquinha, Natalie H. Valentin, Garrett B. Hubbard, Guowei Yin, Henrik G. Dohlman, Sharon L. Campbell

**Affiliations:** 1 Department of Biochemistry & Biophysics, University of North Carolina at Chapel Hill, Chapel Hill, NC, USA; 2 Department of Pharmacology, University of North Carolina at Chapel Hill, Chapel Hill, NC 27599, USA; 3 The Seventh Affiliated Hospital of Sun Yat-sen University, Shenzhen, 518107, China; 4 Lineberger Comprehensive Cancer Center, University of North Carolina at Chapel Hill, Chapel Hill, NC, USA

## Abstract

Heterotrimeric G proteins (Gα, Gβ and Gγ) act downstream of G-protein-coupled receptors (GPCRs) to mediate signaling pathways that regulate various physiological processes and human disease conditions. Previously, human Gαi and its yeast homolog Gpa1 have been reported to function as intracellular pH sensors, yet the pH sensing capabilities of Gαi and the underlying mechanism remain to be established. Herein, we identify a pH sensing network within Gαi, and evaluate the consequences of pH modulation on the structure and stability of the G-protein. We find that changes over the physiological pH range significantly alter the structure and stability of Gαi-GDP, with the protein undergoing a disorder-to-order transition as the pH is raised from 6.8 to 7.5. Further, we find that modulation of intracellular pH in HEK293 cells regulates Gαi-Gβγ release. Identification of key residues in the pH-sensing network allowed the generation of low pH mimetics that attenuate Gαi-Gβγ release. Our findings, taken together, indicate that pH-dependent structural changes in Gαi alter the agonist-mediated Gβγ dissociation necessary for proper signaling.

## Introduction

Within the complex milieu of living cells, intracellular pH (pH_i_) is maintained within a narrow range, as even small changes in pH can affect a myriad of cellular processes including membrane potential, ion transport, cellular growth and metabolism^1,2,3^. Unsurprisingly, disruptions in pH regulation can contribute to the development of pathological conditions such as ischemic heart disease, cancer, and neurological disorders^3,4^. While various proton pumps and transporters play a role in regulating the flow of protons across the membrane to uphold pH_i_ homeostasis, there are intracellular proteins (termed pH sensors) that sense and transmit pH signals, thus orchestrating the regulation of biochemical processes. Among these pH sensors are signal-transducing proteins. Notably, a study by Isom *et. al.* (2013) provided evidence that a subset of signal-transducing heterotrimeric G proteins may serve as intracellular pH sensors^3^. These membrane-associated proteins form a heterotrimeric complex (Gα, Gβ and Gγ) and act downstream of G-protein coupled receptors (GPCRs). GPCRs represent the most extensive group of membrane proteins that are targeted by approved drugs. They play a crucial role in orchestrating the majority of cellular responses to hormones and neurotransmitters through several signaling pathways mediated by different isoforms of G protein (Gαi, Gαs, Gα12/13, and Gαq) that receive and transduce signals through diverse pathways^5,6^. The Gβ and Gγ subunits associate to form a Gβγ heterodimer^3^ whereas the Gα subunit binds GDP or GTP and catalyzes GTP hydrolysis. The Gα subunit is comprised of two distinct domains: a helical domain and a Ras-like domain. Within the Ras-like domain, there are three key ‘Switch’ regions, namely SW-I, SW-II, and SW-III, which play an essential role in its activity (Fig. 2D). In the GDP-bound state, which is an inactive form of Gα protein, these Switch regions exhibit dynamic behavior. In contrast, in the GTP-bound state (the active form of Gα), they become more structured and less dynamic^7^. In the GDP-bound state, the Gα subunit is associated with the Gβγ complex. Upon GPCR stimulated Gα GTP-loading, the Gβγ subunits dissociate from Gα-GTP and along with Gα, promote activation of downstream signaling pathways.

Isom *et. al.* (2013) developed a computer algorithm, pHinder, which predicted that both the mammalian Gαi isoform and yeast homolog Gpa1 contain a core of residues between the Ras-like and helical domains that may promote pH sensing properties^8^. In support of this prediction, both proteins showed pH-dependent changes in thermostability over a pH range from 5.5 to 8. They also found that Gpa1 undergoes phosphorylation under acidic conditions to attenuate pheromone-dependent stimulation of mitogen-activated protein kinases in the yeast^4^. While these findings, taken together suggest that mammalian Gαi may function as a pH sensor, the molecular mechanism, and the biological consequence of pH sensing through Gαi remain unknown.

To expand on past observations, we characterized pH-dependent biochemical and structural/dynamic properties of GDP-bound Gαi, identified the underlying pH-dependent electrostatic network, and determined functional consequences on cellular Gαi activity. We find that the structure, stability, and dynamics of Gαi in the GDP-bound state are highly dependent on pH over the physiological pH range due to a pH-responsive network within the Ras-like domain. These findings differ from a previous report where larger pH-dependent changes in thermostability were observed for the GTP-bound state with ionizable residues predicted to lie at the interface between the Ras-like and helical domains^3^. Our NMR, biophysical and computational analyses indicate that changes in the ionization state of residues within the pH sensing network promote a disorder-to-order transition in Gαi over the physiological pH range, to populate a less ordered state that enhances Gαi-Gβγ association in HEK293 cells at the lower end of the physiological pH range. Identification of the pH-sensing network allowed for the identification and generation of low pH mimetics that reduce pH-dependent Gβγ release from the Gαi-Gβγ complex. Of note, the Gαi Switch III region plays a key role in pH sensing, providing a new role of this understudied Switch region in agonist-mediated Gβγ release, a key important step for both Gαi and Gβγ activation. Taken together, our studies indicate that Gαi undergoes a pH-dependent order-to-disorder transition that modulates Gαi-Gβγ interactions and Gαi activation over the physiological pH range.

## Materials and methods

### Plasmid constructs

A bacterial pET-SUMO vector containing the human Gαi1 gene (GenBank accession no. BC026326) with the first 31 amino acids deleted, N-terminal 6xHis, and SUMO-tag were employed for *in vitro* analyses. Full-length human Gα containing Renilla luciferase (Gα-RLuc8), Gβ and Gγ containing green fluorescence protein 2 (Gγ-GFP2), and neurotensin receptor (NTR) constructs were employed for cell-based BRET assays^4,8^.

### Site-directed mutagenesis

Gαi variants were generated using the Q5 Site-Directed Mutagenesis kit (NEB). Polymerase chain reaction (PCR) primers were designed using the NEBaseChanger (https://nebasechanger.neb.com), an online mutagenesis primer design tool powered by New England Biolabs from Eton Bioscience Inc. Mutagenesis was performed as described^4^. The variant constructs were sequenced by Sanger Sequencing (Genewiz) to confirm successful mutagenesis. Sequencing results for Gαi variants were aligned with published sequences of wildtype (WT) Gαi using Clustal Omega (European Molecular Biology Laboratory, Cambridgeshire, UK).

### Expression and purification of WT Gαi and its variants

Gαi proteins were overexpressed in *E. coli* Agilent BL21-codon plus (DE3)-RP-X competent cells. Cells were grown at 37°C for 3–4 hours to achieve an optical density at 600 nm (OD 600) of 0.60 and then induced with 500 μM isopropylthio-β-galactoside (IPTG). The temperature was then reduced to 18°C and the cultures were left to grow overnight. After 20 hours, bacteria cells were harvested by centrifugation at 13,000 rpm and then resuspended in 50 mL Dialysis buffer (10 mM KH_2_PO_4_, 20 mM K_2_HPO_4_, 0.15 M KCl, 1 mM MgCl_2_, 10 μM GDP, 1 mM β-mercaptoethanol (BME), pH 7.0). Resuspended cells were lysed using a sonicator (Fisher Scientific, #CL-334) with an amplitude of 80 and 5 seconds on/off time ratio for 10 mins/L cells. Clarified lysate was passed through the Ni-NTA column equilibrated with 1 column volume (CV) (50 ml) of Dialysis buffer, washed with Wash buffer (10 mM KH_2_PO_4_, 20 mM K_2_HPO_4_, 0.15 M KCl, 1 mM MgCl_2_, 10 μM GDP, 1 mM BME, 30 mM imidazole, pH 7.0) and eluted using the Elution buffer (10 mM KH_2_PO_4_, 20 mM K_2_HPO_4_, 0.15 M KCl, 5 mM MgCl_2_, 10 μM GDP, 1 mM BME, 250 mM imidazole, pH 7.0). The elution fraction was dialyzed overnight in the presence of a ubiquitin-like-specific protease 1 (ULP1) to cleave the SUMO tag on Gαi. After dialysis, the protein was again passed over a Ni-NTA column equilibrated with 3 CVs of water and 1 CV dialysis buffer. The protein flow-through was further purified by gel filtration chromatography using a Fast Protein Liquid Chromatography (FPLC) system (Äkta Primeplus) and Superdex 75 column (Cytiva). The purity of the protein was verified by SDS PAGE gel electrophoresis, and the concentration of the purified protein was estimated by Thermo Scientific NanoDrop.

### Circular Dichroism (CD) analyses

Gαi proteins were exchanged in CD buffer (10 mM K_2_HPO_4_, 500 μM MgSO_4_, 500 μM tris(2-carboxyethyl)phosphine (TCEP)) using an Amicon centrifugal filter unit (MilliporeSigma, #UFC901024) at 3800 × g, diluted to 15 μM (pH 6.0, 6.4, 6.8, 7.2, or 7.6) and centrifuged (14,000 rpm) for 10 mins at 4 °C. Temperature dependent CD experiments were performed on a Jasco J815CD spectrometer using 15 μM WT or Gαi variants protein in a 1 mm path-length quartz cuvette (Hellma Analytics). Thermal melts were obtained at 222 nm, over a temperature range of 20–95°C, using a temperature increment of 1 C/min. The CD spectral scans were collected for Gαi proteins at different fixed temperatures (e.g. 45°C, 55°C and 65°C) by taking CD measurements every 1 nm from 200–250 nm. Secondary structure was evaluated using the online server BeStSel (Beta Structure Selection)^9^.

### Intrinsic tryptophan fluorescence

Intrinsic tryptophan fluorescence assays were conducted using 2 μM purified WT or variant GDP-loaded Gαi proteins in 200 μl of assay buffer (20 mM HEPES, 50 mM NaCl, 5 mM MgCl_2_, 2 mM DTT) at different pH (pH 5, 6 and 7.2) in the 96 well plate. Gα proteins were excited at 290 nm and intrinsic tryptophan fluorescence was measured from 300 to 400 nm wavelength using a SpectraMax M4 Series Microplate Reader.

### 2D NMR experiments

Triple labeled ^2^H/^13^C/^15^N Gαi samples were generated by expressing Gαi proteins in BL21(DE3)-RIPL *E. coli* cells and growing in minimal medium supplemented with 1 g/L ^15^NH_4_Cl, 3 g/L ^13^C-glucose, 99% D_2_O (Cambridge Isotope Labs)^7^, and purified as described above. Triple labeled Gαi was prepared in 20 mM potassium phosphate (pH=7.0), 50 mM potassium chloride, 5 mM DTT (dithiothreitol), 5 mM magnesium chloride, and 50 μM GDP containing 5% (v/v) D_2_O. For ^15^N-enriched WT and Gαi variants, bacterial expressed proteins were grown in minimal media containing 1 g/L ^15^NH_4_Cl as the sole nitrogen source and purified as described above.

2D NMR ^1^H-^15^N HSQC spectra of Gαi were acquired on a Bruker Avance 850 MHz (14.1 T field strength) NMR spectrometer at 25 °C, with a cryogenic (TCI) 5 mm triple resonance probe equipped with a z-axis gradient. The ^1^H-^15^N HSQC spectra of Gαi-GDP were assigned using a combination of triple resonance experiments, including 3D HNCA, HN(CO)CA, HN(CA)CO and HNCO^7^. TROSY-based pulse sequences were used for sensitivity enhancement. Bruker-TopSpin was used to process the NMR data and NMRFAM-SPARKY was used to visualize and analyze the NMR spectra^10^. For assignments, BMRB 30078 was used as reference spectrum for Gαi-GDP^11^. pH titration studies were performed by calculating the chemical shift perturbation (CSP) of Gαi-GDP over a pH range from 6.4, 6.8, 7.0, 7.2, 7.4 and 7.6. Average ^1^H-^15^N CSP were determined using the formula Δδ = [(Δ^1^HN)^2^ + (Δ^15^N/5)^2^]^0.5^, as previously described^12^. PyMOL (https://pymol.org/2/) was used to generate all images of molecular structures.

### Guanine nucleotide dissociation assay

Gαi proteins were exchanged into nucleotide association buffer (20 mM HEPES, 50 mM NaCl, 5 mM MgCl_2_, 2 mM DTT) using an amicon concentrator (3,800 g, 15 min, 3 rounds). In the cuvette, 0.75 μM (2’-(or-3’)-O-(N-Methylanthraniloyl) Guanosine 5’-Diphosphate, Disodium Salt (Mant-GDP, purchased from Thermofisher) was added to 1 ml of association buffer for the assay. The intrinsic tryptophan (W211) was excited at 280 nm and the Mant-GDP fluorescence intensity at 425 nm was measured as a function of time using a PerkinElmer LS55 luminescence spectrometer. After collecting data for a few seconds to obtain baseline fluorescence, purified 1 μM Gαi-GDP was added to the solution to initiate GDP association. Once the fluorescence reached saturation, 10 × (7.5 μM) GDP was added to the solution to initiate the GDP dissociation.

### Molecular Dynamics (MD) simulations

Gαi-GDP coordinates were extracted from the Gαi-Gβγ complex structure (PDB: 1GP2) to visualize Switch residues not observable in Gαi-GDP structures ^13^. Modeller 9v21.2 was employed to relocate missing residues and generate charged and uncharged states for residues E236, D237 and E245^14^ to represent the charged state as higher pH (above 7.2) and the uncharged state as low pH (below 6.4). Following side-chain optimizations, minimum energy conformations of charged and uncharged GDP-bound Gαi states were identified. Subsequently, MD simulations were performed to investigate conformational and dynamic differences compared to WT GDP-bound Gαi. The CHARMM36 forcefield was used to parametrize the protein and GDP, and simulations were run using GROMACS-2020.3.5^15^. Gαi proteins were solvated in a water cubic box containing approximately 22,500 TIP3P water molecules and maintained a salt concentration of 150 mM through the addition of an appropriate number of Na+ and Cl− ions. The solvated system was energy minimized using the conjugate gradient algorithm and subsequently equilibrated using the V-rescale thermostat and the Parrinello-Rahman barostat^16^. Long-range electrostatic interactions were evaluated using the Particle-Mesh Ewald sum^17^, and all bonds involving hydrogen atoms were constrained using the LINCS algorithm^18^. Simulations were run for 250 ns and were repeated in triplicate to ensure reproducibility and maintain consistency. Structural figures were generated using PyMOL and graphical plots were created using Grace (http://plasma-gate.weizmann.ac.il/Grace/).

### HADDOCK docking

The HADDOCK online server was used for the computational docking of Gαi with Gβγ^19^. The initial structure of Gαi with E236, D237, and E245 residue side chains in either the charged (higher pH) or uncharged state (lower pH) as described above, was obtained from the averaged structure of 250 ns MD simulations. The Gβγ structure was extracted from the Gαi-Gβγ complex crystal structure (PDB:1GP2). Contact residues between Gαi and Gβγ that form the trimeric complex structure were used as active residues and the passive residues were auto selected by the software. The docking model corresponding to the lowest energy was used for further analysis.

### Cell culture

HEK293T cells were maintained, passaged, and transfected in Dulbecco’s Modified Eagle Medium (DMEM, Gibco) containing 10% sterilized Fetal Bovine Serum (FBS, Gibco), 100 Units/mL penicillin, and 100 μg/mL streptomycin (Gibco-ThermoFisher, Waltham, MA) in a humidified atmosphere at 37°C and 5% CO_2_. Transfection was carried out using a lipid-polymer-based transfection agent (Mirus Bio, MIR 5400, Madison, WI). After transfection, cells were plated in DMEM (Dulbecco’s Modified Eagle Medium) containing 10% FBS (Fetal Bovine Serum), 100 Units/mL penicillin, and 100 μg/mL streptomycin for BRET assays.

### Intracellular pH modulation:

The intracellular pH of HEK293 cells was altered by two different methods. In the first method, HEK293 cells were treated with 1–5 μM of trifluoromethoxycarbonylcyanide phenylhydrazone (FCCP) for 5 min. In the second method, intracellular pH was modulated by incubating HEK293 cells with Hanks’ Balanced Salt Solution (HBSS) buffer at different pH for 15 min. Intracellular pH was quantified using the intracellular pH indicator, 2’,7’-bis-(2-carboxyethyl)-5-(and-6)-carboxyfluorescein-acetoxymethyl ester (BCECF-AM) as described^20^. To convert BCECF-AM fluorescence to the intracellular pH values, HEK293 cells were treated with 20 μM nigericin and a calibration curve for pH_i_ and BCECF-AM fluorescence was generated.

### Bioluminescence Resonance Energy Transfer (BRET) assays

BRET assays were carried out as described by Olsen et al. (2020)^8^. Briefly, HEK293T cells were seeded in six-well dishes in DMEM containing 10% FBS, 100 Units/mL penicillin, and 100 μg/mL streptomycin at a density of 300,000 cells/well and allowed to grow to 80–90% confluency. Cells were then transfected using a 1:1:1:1 DNA ratio of NTR:Gα-RLuc:Gβ:Gγ-GFP2 (1000 ng/construct for six-well dishes). TransIT-2020 (Mirus Biosciences, Madison, WI) was used to complex the DNA at a ratio of 3 μL Transit/μg DNA, in OptiMEM (Gibco-ThermoFisher, Waltham, MA) at a concentration of 1 μg DNA/μL OptiMEM. The next day, cells were harvested from the plate using trypsin (0.25% EDTA, Gibco, #25200056), and plated in poly-D-lysine-coated white, clear bottom 96-well assay plates (Greiner Bio-One, Monroe, NC) in DMEM containing 1% dialyzed FBS, 100 Units/mL penicillin, and 100 μg/mL streptomycin at a density of 50,000 cells/well.

One day after seeding in 96-well assay plates, white backings (Perkin Elmer, Waltham, MA) were applied to the plate bottoms, and the growth medium was aspirated. Cells were washed three times with Assay buffer (1X HBSS, 20 mM HEPES, pH 7.4), then 60 μL of Assay buffer was added immediately, followed by a 10 μL addition of freshly prepared 50 μM coelenterazine 400a (1-bisdeoxycoelenterazine) (Nanolight Technologies, Pinetop, AZ) in each well. After a five-minute equilibration period, cells were treated with 30 μL of 3x neurotensin (3 × 10^−5^M) for another 5 minutes. Plates were then read in a plate reader (Clariostar, BMG Labtech, Ortenberg, Germany) at 395 nm (Rluc8-coelenterazine 400a) and 510 nm (GFP2) with a slit width of 8 nm using 10 flashes per spiral well scan. Plates were read serially five times, and measurements from the third read were used in all analyses. BRET ratios were computed as the ratio of the GFP2 emission to Rluc8 emission.

### Statistical Analysis

CD melt-curves were fit to Boltzmann sigmoidal equation in Prism 9.3.1 (GraphPad Software, San Diego, CA). Concentration-response curves for BRET assays were fit to a three-parameter logistic equation. Raw BRET concentration-response curves were normalized to the best-fit maximum within a data set. BRET data were represented as mean ± SEM. Data analysis was carried out using Prism 9.3.1 (GraphPad Software, San Diego, CA).

## Results

### pH modulation of GDP-bound Gαi structure and dynamic properties

**Fig. 1.**
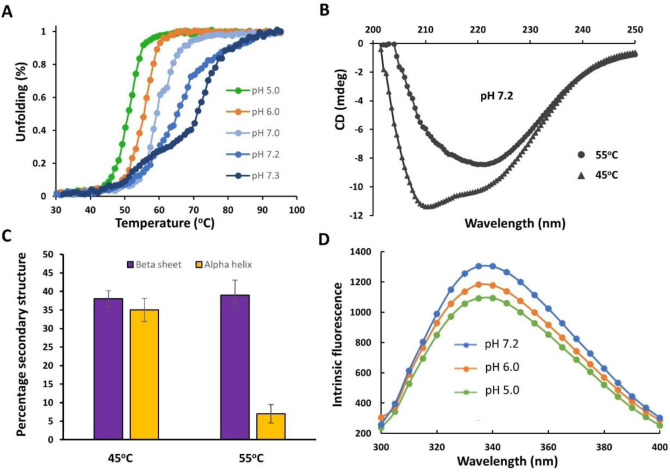
Gαi-GDP thermostability and intrinsic tryptophan fluorescence are pH dependent. (A) Representative CD thermal melt (222 nm, 20–95 °C) of Gαi-GDP (15 μM) shows enhanced thermostability at higher pH (N = 3). (B) Representative CD spectral scans (200–250 nm) of Gαi-GDP collected at 45 and 55°C and pH 7.2 (N = 2). (C) Bar graph derived from temperature-dependent CD spectral scans highlighting the loss of alpha-helical but not beta-sheet secondary structure. (D) Representative intrinsic tryptophan fluorescence spectra of Gαi-GDP (2 μM, excitation = 280 nm, emission = 300 – 400 nm) shows enhanced fluorescence as the pH is increased from 5 to 7.2 (N = 2, performed in triplicate).

Earlier investigations into mammalian Gαi demonstrated changes in structure and stability in response to pH variations^8^. While these findings suggest a potential role for Gαi as a pH sensor^8^, the molecular basis and functional relevance has yet to be established. Herein, we apply comprehensive and multidisciplinary approaches to evaluate how pH changes in the physiological range affect structure, stability and Gαi1 activity in vitro and in cells. We first conducted CD experiments on GDP-bound Gαi to monitor pH-dependent changes in thermal unfolding, stability and secondary structure. Further, to monitor thermal unfolding and stability, we collected CD spectra at 222 nm as a function of pH and temperature. As shown in Fig. 1A, a striking and gradual increase in thermal stability (T_m_ ~ 25 °C) of Gαi-GDP was observed over the pH range from 5 to 7.3. Notably, the thermal unfolding transition for Gαi-GDP appears cooperative at low pH but shifts to a multi-state unfolding transition over the physiological pH range (Fig. 1A). Gαi contains two subdomains, a Ras-like and helical domain. To evaluate differential unfolding at higher pH (above pH 7.1), we conducted CD scans (200–250 nm) for Gαi-GDP as a function of temperature at pH 7.2. As shown in Fig. 1B and C, CD spectra revealed a significant reduction in alpha-helical propensity, but not the beta-sheet propensity as the temperature is raised from 45–55°C. These findings indicate that the helical domain melts first, followed by the Ras-like domain, with the Ras-like domain showing the greatest change in thermal stability at higher pH.

Given the enhanced stability observed at higher pH, we employed intrinsic tryptophan fluorescence experiments using tryptophan (W211) in the SW-II region (Fig. 3A) as a fluorescence probe to monitor differences in solvent exposure as a function of pH which indirectly indicates pH-dependent switch conformational changes. It has previously been shown that the more dynamic and less ordered GDP-bound state of Gαi promotes enhanced exposure of W211 resulting in a fluorescence decrease relative to that of the Gαi-GTP state^7,21^. As shown in Fig. 1D, intrinsic Gαi-GDP tryptophan fluorescence increases over the pH range from 5 to 7.2, suggesting that higher pH promotes decreased solvent accessibility possibly due to enhanced interactions and structural order, consistent with the greatly enhanced stability observed by CD. Taken together, these results support a disorder-to-order transition at higher physiological pH for Gαi in its GDP-bound form.

**Fig. 2:**
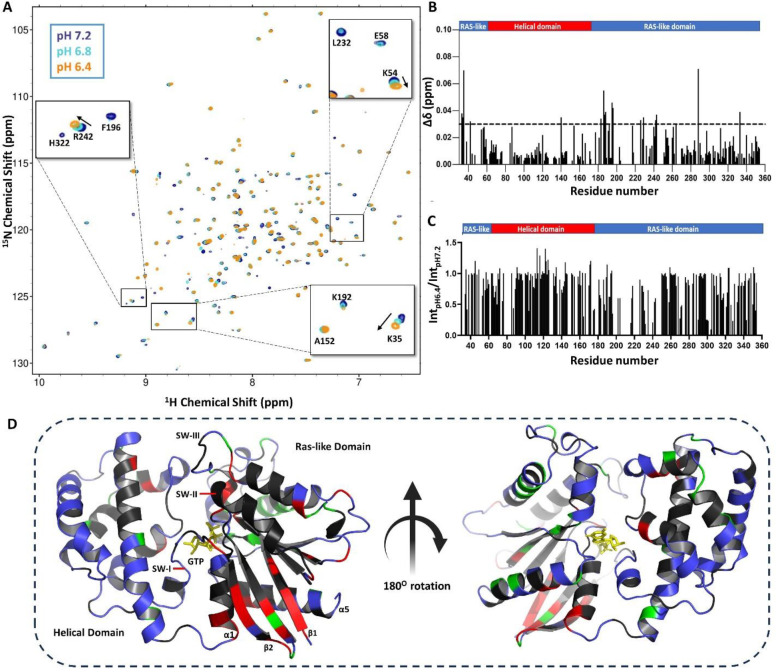
Gαi-GDP ^1^H-^15^N 2D NMR spectral changes as a function of pH. (A) Representative 2D ^1^H-^15^N TROSY-HSQC spectral overlay of ^2^H, ^13^C, ^15^N-enriched Gαi-GDP (230 μM) at pH 6.4, 6.8, and 7.2, highlighting peak shifts and line broadening. Spectra were acquired on a Bruker Avance III 850 MHz instrument at 25 °C (N = 2). (B) Chemical shift perturbation (CSP) and (C) peak intensity ratio (Int_pH 6.4_/Int_pH 7.2_) for pH 6.4 and 7.2. The striped horizontal line represents the mean value of Δδ amide. Most of the residues that show significant CSP, and broadening lie within the Ras-like domain (α1, α5, β1, β2 and β2-β3 loop). (D) Spectral differences are highlighted on a ribbon diagram of GTP-bound Gαi (PDB: 1CIP). NH residues with CSP greater than 0.03 ppm are represented in red. Residues with decreased peak intensity (line broadening) at pH 6.4 relative to pH 7.2 are shown in green. Residues missing or unassigned are shown in black color while unaffected residues are shown in blue color.

To further probe whether a pH-dependent disorder-to-order transition occurs in GDP-bound Gαi, we conducted 2D NMR analyses. For these studies, we prepared ^15^N enriched Gαi-GDP and collected a 2D ^1^H-^15^N Heteronuclear Single Quantum Coherence (HSQC) NMR spectra over a physiologically relevant pH range (6.4 –7.6). Enrichment with ^15^N allows the detection of backbone and sidechain NH resonances within Gαi and provides a site-specific probe for every amino acid except proline. The 2D HSQC overlay of Gαi-GDP at pH 6.4, 6.8 and 7.2 is shown in Fig. 2A, with an HSQC overlay comparing pH 6.4 versus pH 7.6 shown separately in Supplementary Fig. S1. Spectra acquired at lower pH show significant chemical shift changes, line broadening and loss of several peaks in comparison to those obtained at higher pH values, suggesting the protein is more dynamic at lower pH (Fig. 2A). This data correlates well with CD and tryptophan fluorescence data which suggests a less thermostable structure of Gαi-GDP at lower pH. The pH-dependent HSQC changes observed are consistent with an earlier report which showed extensive broadening of NH peaks in the ^1^H-^15^N HSQC spectrum of Gαi-GDP at pH 6 relative to pH 7^22^.

Residue-specific chemical shift perturbation and peak intensity changes associated with changes in pH between 6.4 and 7.2 are plotted in Fig. 2B and C, respectively. Most pH-dependent spectral changes are localized to α1, α5, β1, β2 and the β2-β3 loop within the Ras-like domain as mapped onto the Gαi-GTP structure (PDB:1CIP) in Fig. 2D. Of note, several resonances within these key regions are undetectable in the GDP-bound state at all pH values (Fig. 2D, black) which somewhat limits NMR analyses. These findings are consistent with previous work showing that residues associated with the Switch regions in the GDP-bound state of Gαi exhibit enhanced backbone dynamics and are broadened and undetectable compared to the resonances associated with the GTP-bound state^7^. Taken together, 2D NMR analyses, CD data and tryptophan fluorescence data indicate that the Switch regions of Gαi-GDP are more dynamic and less structured at pH 6.4 – 6.8 compared to pH 7.2 – 7.5.

### pH-dependence of GDP binding to Gαi

**Fig. 3:**
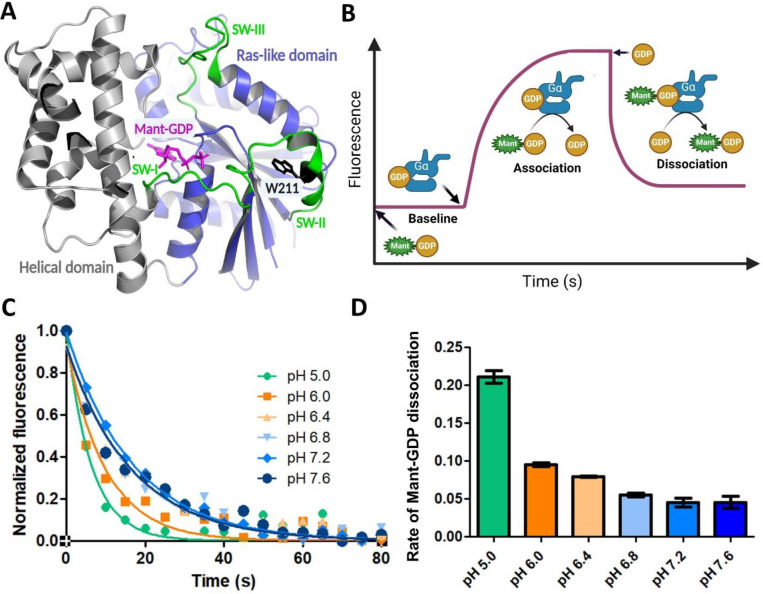
Rates of Gαi GDP nucleotide dissociation vary with pH. (A) Structure of Gαi (PDB:1GP2) highlighting the position of tryptophan (W211) in Switch II and bound Mant-GDP. (B) Diagram displaying experimental setup of FRET-based determination of Gαi nucleotide association and dissociation rates. (C) Rate of GDP dissociation from Gαi as a function of pH by monitoring the time-dependent decrease in FRET emission of Gαi-loaded Mant-GDP at 445 nm upon the addition of 7.5 μM GDP. (D) The rate of Mant-GDP dissociation decreases as the pH increases. Data are averages of two independent experiments performed in duplicate (±SE).

Gαi when bound to GDP, adopts a conformational ensemble and dynamic properties distinct from that of the GTP-bound state^7^. This in turn drives recognition of regulatory factors and downstream targets. Given our findings that pH modulates Gαi-GDP structure, stability and dynamics, we asked if pH could alter GDP binding to Gαi protein. For that purpose, we performed Mant-GDP dissociation assays. For these assays, the rate of GDP dissociation was determined by adding excess GDP to Mant-GDP loaded Gαi and monitoring Mant fluorescence changes (by FRET upon tryptophan excitation) as a function of time at different pH values (Fig. 3A–B). As shown in Fig. 3C–D, small differences in GDP dissociation rates were observed over the pH range (pH 6.8–7.4), indicating that GDP binding is not significantly altered at physiological pH.

### Identification and characterization of pH sensing residues of Gαi-GDP

**Fig. 4:**
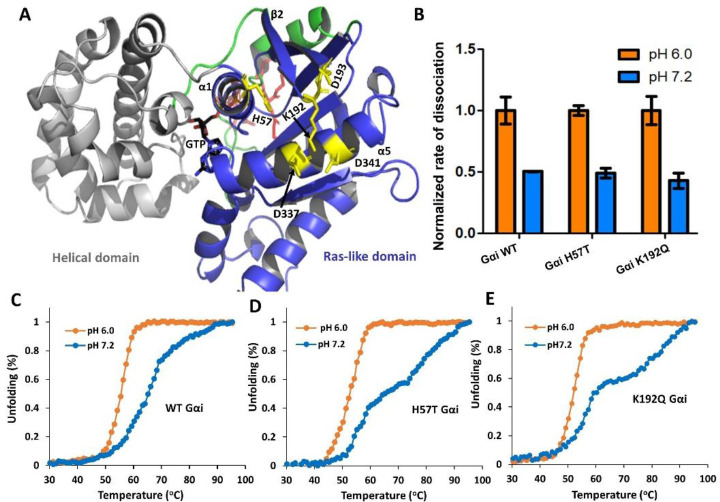
pH-dependent nucleotide exchange and stability of Gαi variants from the GDP release network. (A) Ribbon diagram of GTP-bound Gαi (PDB: 1CIP) highlighting electrostatic network near GDP release network. The helical domain, Ras-like domain and the Switch regions are shown in gray, blue, and green, respectively. Charged residues are shown as yellow sticks. (B) Rates of GDP dissociation is compared for WT Gαi and Gαi variants (H57T and K192Q) as a function of pH. Data are averages of two independent experiments performed in duplicate (±SE) (C) Representative CD melt profile of GDP-bound WT and variant Gαi (N=2), Gαi H57T (D) and K192Q (E) proteins. Gαi release network variants retain pH-dependent thermal unfolding profiles similar to WT Gαi.

**Fig. 5.**
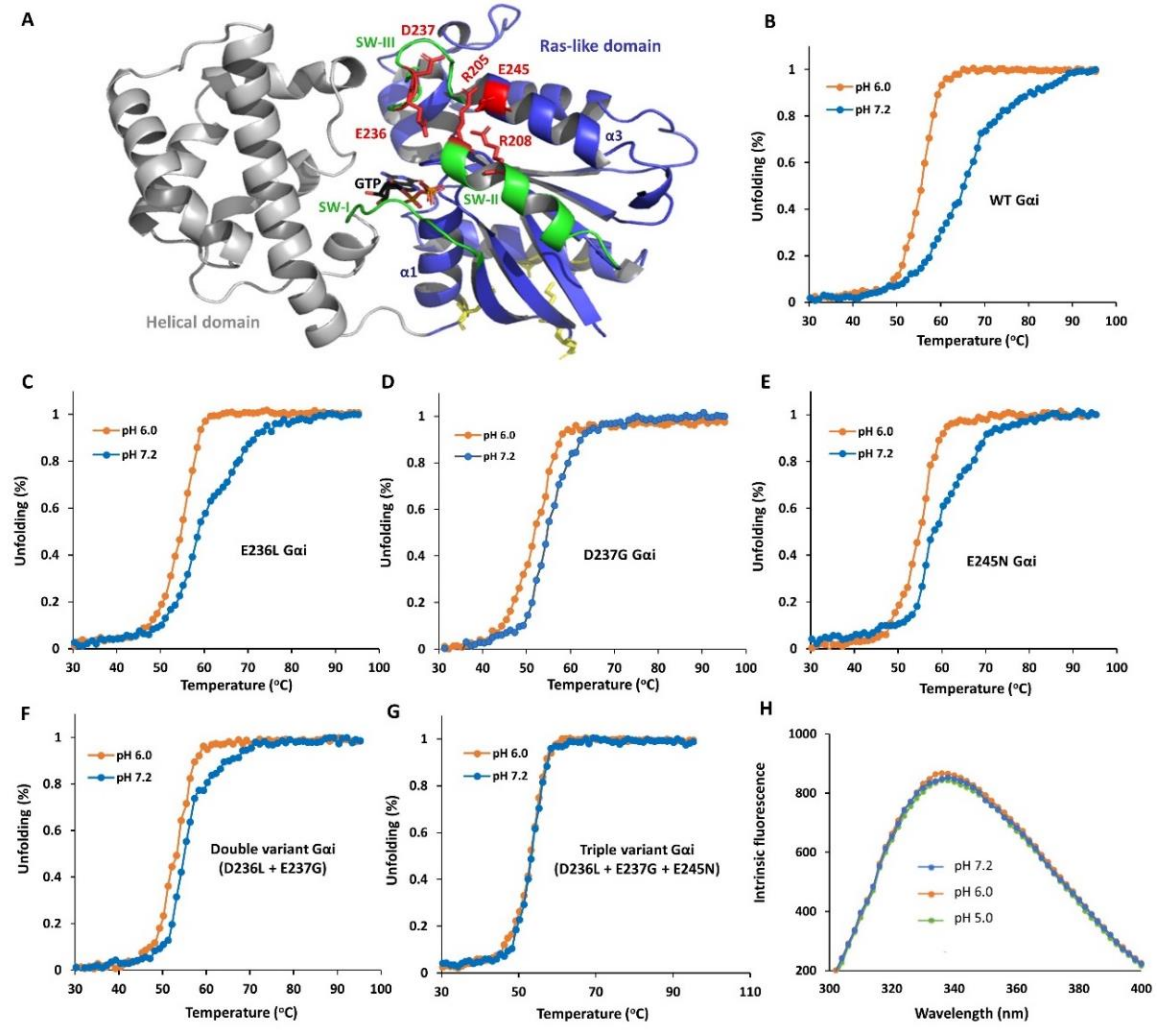
pH-dependent stability and tryptophan fluorescence of Gαi Switch network variants. (A) The putative pH-dependent electrostatic network (red) within the Switch regions (green) is highlighted on the ribbon diagram of GTP-bound Gαi (PDB: 1CIP). (B) Comparison of representative CD melt profiles obtained from two independent experiments at pH 6.0 and 7.2 for WT Gαi-GDP, (C) Gαi-GDP E245N, (D) Gαi-GDP E236L, (E) Gαi-GDP D237G (F) Gαi-GDP double variant (E236L/D237G), and (G) a Gαi-GDP triple variant (E236L/D237G/E245Q). The CD thermal profile for Gαi single and double variants shows decreased pH-dependent thermal stability while the triple variant shows a complete loss of pH-dependent thermal stability compared to WT Gαi. (H) Representative intrinsic tryptophan fluorescence spectra of Gαi-GDP triple variant (2 μM, excitation = 280 nm, emission = 300 – 400 nm) as a function of pH (N = 2, performed in triplicate). Consistent with the CD results, pH-dependent intrinsic tryptophan (W211) fluorescence observed for WT Gαi-GDP is abolished for the Gαi triple variant supporting a role for E236, D237 and E245 in pH-dependent stability and structural changes.

To elucidate the molecular basis for pH-dependent stability and conformational dynamic changes in GDP-bound Gαi, we sought to identify key residues involved in pH sensing. As extensive broadening of resonances in GDP-bound spectra prevented pKa determination by NMR, we examined and identified two networks of charged residues in regions, designated as the ‘GDP release network’ (Fig. 4A) and ‘Switch network’ (Fig. 5A), that showed pH-dependent changes in the NMR spectra (Fig. 2D). The GDP release network contains residues within α1, α5, β2, and β3, and was previously shown to be important for GDP release during the GPCR-mediated activation of the Gαi^23^. We postulated that if this network plays a key role in pH sensing, mutation of charged residues within this network (e.g., H57, H188, K192, D193, H195 and D337) would reduce pH-dependent Gαi thermostability, due to protonation and loss of electrostatic interactions that destabilize Gαi tertiary structure.

To examine whether this network modulates pH-dependent changes in stability and nucleotide binding, we generated several Gαi variants and conducted pH-dependent CD thermal melt and nucleotide dissociation assays. To select neutral or uncharged amino acid substitutions that retain or have minimal effect on Gαi structure, we employed the Rosetta modeling suite. Rosetta replaces a desired amino acid within the protein to all possible amino acid substitutions and predicts associated free energy changes^24^. Substitutions that minimally perturb free energy are predicted to retain protein structure. Using this strategy, we identified four variants (H57T, H188V, K192Q and H195N) predicted to retain Gαi structure and stability. To evaluate pH-dependent thermostability associated with these Gαi GDP release network variants, we acquired CD thermal melts at pH 6 and 7.2, as shown in Fig. 4. Of note, all four variants retained pH-dependent thermostability similar to WT Gαi (data not shown for H188 and H195) (Fig. 4C–E). We also performed GDP dissociation assays to evaluate whether the variants alter nucleotide binding (Fig. 4B). All variants showed pH-dependent GDP dissociation rates similar to WT Gαi. These findings indicate that the GDP release network does not significantly modulate pH-dependent stability or nucleotide binding (Fig. 4B–F).

As a number of charged residues within the Switch regions form stabilizing electrostatic interactions in the active Gαi-GTP bound state, we next postulated that the decreased stability of Gαi-GDP at lower pH may result from protonation of pH-dependent Switch network (SW-I, SW-II, SW-III and α3) residues. To test this hypothesis, we selected residues from the Switch network shown or predicted to be important for Switch stability in the GTP-bound form of Gαi^25^. Then, based on Rosetta prediction, we mutated a subset of charged residues within this network (e.g. R205N, R208Q, E236L, D237G, R242Q and E245N) predicted to least perturb Gαi structure (Fig. 5A). As shown in Fig. 5B–E, two of the variants located in SW-III (E236L and D237G) and α3 (E245N), respectively, showed a reduction in pH-dependent thermostability between pH 6 and 7.2 relative to WT Gαi. Moreover, a ‘double variant’ consisting of two substitutions (E236L and E237G) from SW-III showed further reduction in pH-induced stability but not complete abolishment of pH dependence (Fig. 5F). Yet, a ‘triple variant’ containing all three substitutions (Gαi E236L, D237G and E245N) effectively eliminated pH-induced stability changes relative to WT Gαi (Fig. 5G), suggesting a key role for these three Switch network residues (E236, D237 and E245) in stabilizing Gαi at higher pH. Other variants from the Switch network at R205 and R208 did not show any change in pH-dependent thermostability in comparison to WT Gαi protein (Supplementary Fig. S2). We also monitored tryptophan fluorescence of the GDP-bound Gαi triple variant as a function of pH to examine changes in solvent accessibility of SW-II residue W211. Consistent with the loss in pH-dependent thermal stability, we found that the double variant and triple variant reduce and abolish (Supplementary Fig. S3) pH-dependent fluorescence intensity changes relative to WT Gαi-GDP, respectively (Fig. 5H). As the CD thermal and fluorescence profiles associated with the double variant and triple variant mimic WT Gαi at lower pH (pH 6), we refer to these variants as “low pH mimetics”. Further to confirm that the identified residues participate in pH sensing, we performed NMR analyses for the double variant at pH 6.4 and pH 7.2 in comparison to WT GDP-bound Gαi. As shown in Supplementary Fig. S4, pH-dependent changes in peak intensity and chemical shift perturbations are significantly reduced in the double variant with respect to WT Gαi. Taken together, our results point to key residues in the Switch network that regulate pH-dependent stability and conformational dynamic properties.

### MD simulations of pH-dependent Gαi-GDP conformational dynamics

We identified three residues in the GDP-bound Gαi, including two SW-III residues (E236, D237) and one α3 residue (E245), that appear to play a key role in pH-dependent stability and conformational dynamics. To evaluate how these residues form pH-dependent electrostatic interactions that stabilize the Switch regions at higher pH, we employed MD simulations. We generated Gαi structures using the Gαi-GDP crystal structure (PDB: 1GP2) as a starting point and then changed the protonation state of side chains associated with residues E236, D237 and E245 to simulate a low pH and high pH state, respectively.

**Fig. 6:**
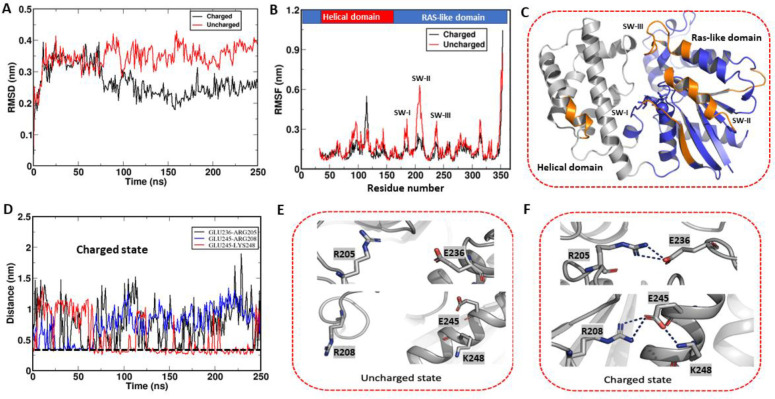
MD simulations of WT Gαi-GDP highlighting uncharged and charged states of E236, D237 and E245. To mimic the low pH state of Gαi-GDP (uncharged state), E236, D237 and E245 side chains were protonated using the Gαi-GDP crystal structure (PDB: 1GP2). MD simulations of Gαi-GDP in both charged (deprotonated) and uncharged states (protonated) were performed for 250 ns. (A) Root means square deviation (RMSD) of Gαi Cα atoms in charged (black) and uncharged (red) states, as estimated by superposing each frame of the trajectory to the corresponding starting structure. Gαi-GDP shows higher RMSD in the uncharged state indicative of enhanced dynamics consistent with NMR at lower pH. (B) Residue-wise root mean square fluctuation (RMSF) derived from MD trajectory (100 ns) of charged (black) and uncharged (red) Gαi-GDP, highlights enhanced Switch dynamics in the uncharged state. (C) Mapping of residues that display higher RMSF (orange) on the Gαi-GTP crystal structure (1CIP) indicates that the SW-I, SW-II and SW-III become more dynamic in the uncharged state compared to the charged state, consistent with the reduced thermal stability and intrinsic tryptophan fluorescence at lower pH. (D) Time evolution of Gαi -GDP salt-bridge formation associated with charged trajectory shows three salt-bridges in the charged state but not in the uncharged state of Gαi. The cut-off value of salt-bridge distance = 3.2 Å is shown as a dotted line. (E) Representative conformation of Gαi-GDP in the uncharged (protonated) state extracted from the MD trajectory shows a lack of salt-bridge interactions due to the destabilization of the SW-III region. (F) Zoomed section depicting salt-bridge interactions extracted from the corresponding MD trajectory (E236-R205, E245-R208, and E245-K248) for the average conformation of charged Gαi-GDP.

Analysis of MD simulation trajectories of GDP-bound Gαi collected for 250 ns in charged versus uncharged states revealed higher root mean square deviation (RMSD) in the protonated or uncharged state. As shown in Fig. 6A, the RMSD of the uncharged state indicated higher dynamics, with RMSD values around 0.4 nm, while the charged state exhibited lower RMSD values of approximately 0.25 nm. Based on analyses of the MD trajectories, we attribute the RMSD reduction associated with the charged state to the formation of salt-bridge interactions within the Switch regions. To further probe the dynamic properties of the Gαi-GDP Switch regions, we calculated residue-specific root mean square fluctuation (RMSF) in the uncharged state (Fig. 6B and C) and mapped RMSF changes onto the Gαi-GTP crystal structure (PDB:1CIP). As shown in Fig. 6B and C, our simulations suggest increased dynamics of SW-I, SW-II, and SW-III in the uncharged state compared to the charged state, consistent with reduced thermal stability at lower pH. Overall, these findings suggest that at lower pH (pH 6.4–7), there is a loss of electrostatic interactions within the Switch regions of GDP-Gαi that promotes enhanced dynamics, consistent with observations from NMR and CD data.

Representative snapshots extracted from the MD trajectories of Gαi-GDP provide insights into the dynamic behavior of Gαi as a function of pH. In the charged or higher pH state of Gαi-GDP, we observe the transient formation of three critical salt-bridge interactions: E236 interacting with R205, E245 with R208, and E245 with K248 (Fig. 6D and F). Notably, these residues form electrostatic interactions as observed in crystal structures of active Gαi-GTP state (PDB:1CIP) and play a pivotal role in the stabilization of the Switch regions. In particular, E236 and D237 side chains from SW-III interact with R205 and R208 in SW-II whereas α3 residue E245 interacts with R208^25^. In the uncharged state of Gαi-GDP (lower pH), conformations extracted from MD simulation trajectories exhibit a notable absence of these salt-bridge interactions (Fig. 6E), which we attribute to the destabilization of the SW-III and SW-II region (Fig. 6E). As a result, the protein displays increased dynamics, as evidenced by higher RMSD and RMSF values, suggesting greater structural fluctuations. The protonation of key residues interferes with the formation of electrostatic interactions leading to their destabilization. Consequently, the protein becomes more dynamic and less thermally stable under more acidic conditions.

To predict how a pH-dependent disorder-to-order transition in Gαi-GDP affects the interaction of Gαi with binding partners, we analyzed interactions with GPCRs or Gβγ. Agonist-simulated GPCRs interact with Gαi-GDP through α5, which is part of the GDP release network. As inspection of the crystal structure of β1-adrenergic receptor with Gαi and Gβγ (PDB: 7S0F) indicates GPCR engagement does not alter the structure of Switch II and III^26^, we predict that pH changes over the physiological pH range do not significantly modulate GPCR interactions with Gαi. Conversely, Gβγ binds to Gαi-GDP through the SW-II region which is more dynamic in the GDP-bound form than it is in the GTP-bound state. We propose, consistent with a recent study^25^, that residues from the Switch network (including the three identified pH sensing residues) provide stabilization of SW-II in the GTP-bound form of Gαi which in turn, prevents interaction with Gβγ. Since our MD data show enhanced dynamics in the protonated or lower pH state of Gαi-GDP, we predict that at the lower end of the physiological pH range, Gαi engages Gβγ with higher affinity resulting in downregulation of Gαi and Gβγ mediated downstream signaling (Fig. 8). To evaluate this hypothesis computationally, we performed HADDOCK docking of Gαi-GDP with Gβγ in both deprotonated charged and protonated uncharged states. The Gαi structures in the charged and uncharged states were obtained as described above, while the Gβγ structure was extracted from the Gαi-Gβγ trimeric complex crystal structure (PDB: 1GP2). The HADDOCK docking score, which is a weighted sum of a variety of energy terms (including van der Waals, electrostatic, desolvation, and restraint violation), was calculated to be −147 and −203 for the charged and uncharged states, respectively. Evaluation of this docking score suggested higher affinity binding of Gαi with Gβγ in the uncharged state due to additional polar contacts between Gαi and Gβγ, implying that low pH enhances the binding of Gαi to Gβγ. Taken together, our computational analyses suggest that Gαi-Gβγ interactions are modulated by pH.

### Analyses of pH-dependent Gαi-Gβγ interactions

**Fig. 7:**
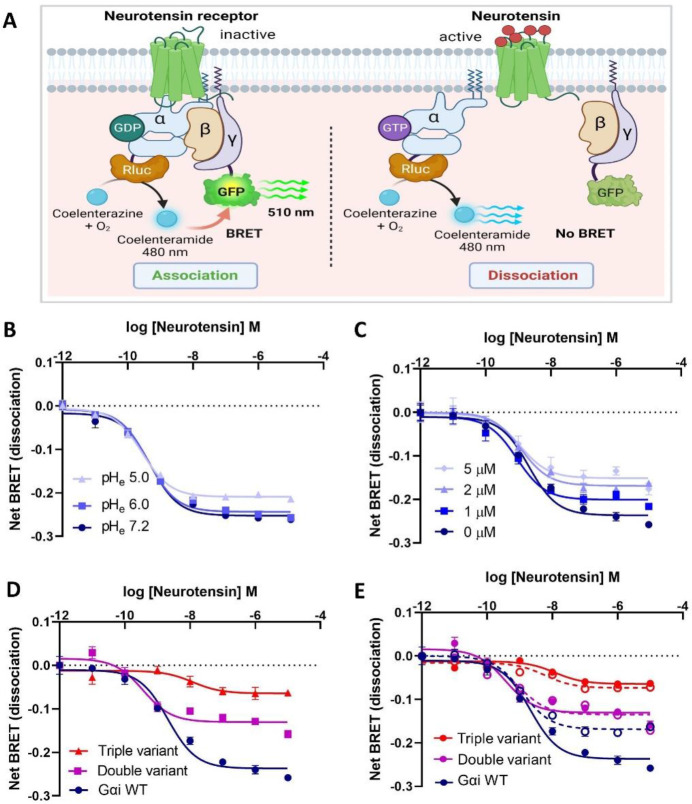
BRET assay showing enhanced Gαi-Gβγ association at lower intracellular pH for the Gαi Switch variants. (A) Schematic diagram (BioRender) illustrating the BRET assay used to monitor receptor-mediated dissociation of Gαi from Gβγ. For this assay, the GPCR neurotensin receptor, Gα Renilla luciferase 8 (Rluc8), Gβ3 and γ9-GFP fusion constructs were co-transfected in HEK29T cells and fluorescence (510 nm) monitored after addition of the substrate and agonist (neurotensin). Net BRET (dissociation) plotted as γ9-GFP (acceptor) over Gα-Rluc (donor) ratio as a function of log doses of neurotensin. Neurotensin receptor-mediated BRET shows that lowering pHi (from 7.2 to 6.7) either by altering extracellular pH (B) or by the addition of FCCP (C), decreases Gαi dissociation from Gβγ in HEK29T cells. (D) WT Gαi shows significantly higher dissociation from Gβγ relative to both double variant and triple variants (low pH mimetics of WT Gαi). (E) The difference in net BRET signal as a function of FCCP concentration (0 μM in solid line and 2 μM in dotted line) for double variant and triple variants is reduced with respect to WT Gαi, confirming key roles for E236, D237, and E245 in pH-mediated Gβγ dissociation for Gαi-Gβγ complex. All BRET data are averages of two independent experiments performed in duplicate (±SE).

**Fig. 8:**
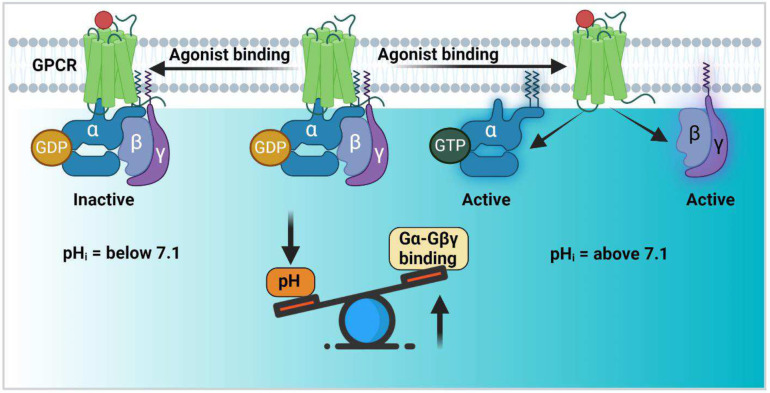
Proposed mechanism of Gαi pH regulation. Activation of Gαi by GPCRs leads to the release of Gαi from Gβγ and stimulation of signaling pathways. A distinct mechanism of signaling regulation may occur by intracellular pH regulation. Gαi protein may act as intracellular pH sensors to regulate Gαi-Gβγ interactions with lower pHi enhancing Gαi-Gβγ interactions to inhibit Gαi signaling.

Gβγ interacts with the dynamic SW-II region of Gαi in the GDP-bound state. Upon upstream activation by GPCRs, Gαi becomes activated through the exchange of GDP for GTP. In the GTP-bound state, the SW-II region becomes rigid and Gαi is unable to adopt a conformation compatible with binding to Gβγ. Given our findings that Gαi adopts a more dynamic state at the lower end of the physiological pH range (6.8–7), we hypothesized that Gαi-GDP engages Gβγ with a higher affinity at lower pH, whereas higher pH (7–7.5) promotes Gβγ release from Gαi-GDP. Consistent with this hypothesis, our MD analyses indicate that pH-mediated changes in the protonation state of key Switch residues alter electrostatic interactions and the dynamic properties of Switch regions of Gαi in its GDP-bound form which may, in turn, modulate Gαi-Gβγ interactions and thus the downstream signaling (Fig. 8). To experimentally evaluate the pH dependence of Gαi-Gβγ interactions in a cellular context, we conducted bioluminescence resonance energy transfer (BRET) assays in human HEK293T cells. For these assays, WT Gαi was tagged with Renilla luciferase (RLuc) and co-transfected with Gγ tagged with GFP, Gβ and the neurotensin receptor. Upon stimulation with neurotensin (agonist), the Gα-RLuc (energy donor) and Gβγ−GFP (energy acceptor) dissociate, with receptor-catalyzed dissociation of the Gαi-Gβγ complex measured by comparing energy transfer from donor to acceptor (Fig. 7A). To directly evaluate whether lower intracellular pH (pH_i_) reduces Gαi-Gβγ dissociation, we employed two distinct approaches to alter and monitor pH_i_ changes in HEK293 cells. Intracellular pH was determined by treating HEK293 cells with BCECF dye and determining the ratio of emission intensity (detected at 535 nm) when the dye is excited at ~490 nm and ~440 nm^20^. To convert the fluorescence ratios to pH_i_, a calibration curve of HEK293 cells treated with 5 μM nigericin was generated (Supplementary Fig. S5A). Treatment of cells with nigericin makes the pH_i_ equivalent to extracellular pH (pH_e_) thus the calibration curve for pH_e_ vs. fluorescence can be used to convert fluorescence into cellular pH_i_. To alter the pH_i_, we compared results using two different strategies. One common method to reduce pH_i_ is the addition of Carbonyl cyanide 4-(trifluoromethoxy) phenylhydrazone (FCCP), an uncoupler of oxidative phosphorylation in the mitochondria^27^. We determined that cells treated with 1 μM FCCP generated a pH_i_ of ~7.04, while at 5 μM the pH_i_ was further reduced to 6.85 (Supplementary Fig. S5B). As one drawback of FCCP is that it can alter mitochondrial energetics, we employed a second method to alter pH_i_ by modulating extracellular pH. As shown in Supplementary Fig. S5C, lowering extracellular pH to 6 and 5, caused a reduction in pH_i_ from 6.9 and 6.7, respectively which is consistent with a previous report^28^. To evaluate the pH dependence of Gαi-Gβγ dissociation in cells, we conducted the BRET assay by expressing WT Gαi in HEK293 cells and inducing acidosis in the cytoplasm by either altering extracellular pH (Fig. 7B) or by the addition of varying concentrations of FCCP (Fig. 7C). Results obtained from the BRET assays show that lowering pH_i_ (pH_i_ 7.2– 6.85 by FCCP or 7.2–6.7 by changing pH_e_) reduces agonist-mediated Gβγ release from Gαi, indicating that Gαi-Gβγ interactions are highly dependent on pH over the physiological pH range.

Further to confirm that the modulation of Gβγ release by pH occurs through the identified 3 residues of Gαi, we performed the BRET assay using the double or triple Gαi variants. As shown in Fig. 7D, agonist-stimulated Gαi dissociation from Gβγ is significantly reduced for the “double variant” and further reduced for the triple variant with respect to WT Gαi. These variants appear to serve as low pH mimetics, as they produce BRET similar to that obtained at lower pH_i_. This observation was further validated by BRET assays performed for double variant and triple variant at different pH_i_. Taken together, these findings suggest that enhanced Gαi-Gβγ association at lower pH is due to the loss of electrostatic interactions within the Switch network needed for Gαi-Gβγ formation.

## Discussion:

The recognition of pH sensors among biomolecules is crucial in understanding cellular processes, as they play a pivotal role in responding to changes in the proton concentration within the physiological range. While many biomolecules experience alterations in their protonation state, only a specific subset serves as pH sensors, exhibiting pH-dependent functional changes that influence cellular processes.

One of the first pH-sensing proteins to be characterized is hemoglobin. In acidic environments, hemoglobin exhibits reduced affinity for oxygen, a phenomenon known as the Bohr effect. This effect is due to the protonation of specific amino acid residues, including histidine 146, located in the α subunit of hemoglobin^29^. The protonation of H146 promotes the formation of a salt bridge with a nearby aspartate residue (D94) on the β subunit. This interaction stabilizes the deoxygenated or T-state conformation of hemoglobin, reducing its affinity for oxygen and facilitating the release of oxygen to tissues where it is needed^30^. After the identification of hemoglobin, proton-sensing ion channels and receptors governing cytosolic pH have been a focal point in research.

More recent structural informatics calculations have shown that buried ionizable networks are a structural hallmark of pH sensitivity^3^, and the large pKa shifts exhibited by buried sidechains can be harnessed by proteins to drive pH-dependent changes in structure, stability, and function. Indeed, networks of buried ionizable amino acids are conserved in nearly all of the 100+ Gα protein structures in the Protein Data Bank (PDB)^3^. On that basis, the cell signaling GTPases, Gαi and its yeast homolog Gpa1, were proposed to function as pH-sensing proteins. The study indicated that the stability of heterotrimeric Gα proteins exhibits a significant dependence on pH levels across a broad range from 5 to 8.0. Furthermore, alterations in yeast intracellular pH (pH_i_) were shown to promote Gpa1 phosphorylation and subsequently dampen the mitogen-activated protein (MAP) kinase signaling pathway^4^. In addition to our findings that Gαi serves as a pH sensor to regulate Gα-Gβγ interactions, recent studies indicate that select GPCRs possess pH sensing properties that modulate extracellular signaling to Gα proteins^31,32,33^. These and other GPCRs may work independently or synergistically with Gαi proteins to transduce extracellular pH-dependent signals to changes in intracellular pH^28,34,35^.

Herein, we find that as pH is raised from 6.8 to 7.3, the thermostability of Gαi-GDP is greatly enhanced (ΔTm ~20°C) due to the formation of an electrostatic network within the Ras-like domain. Since thermostability changes for GDP-bound Gαi were significantly larger (~20°), relative to the GTP-bound form (~8°), we focused on characterizing pH-dependent structural and dynamic changes associated with the GDP-bound form of Gαi in this study. Using pH-dependent NMR analyses and thermal stability profiling on WT and mutant proteins, we identified three key pH-sensing residues that drive pH-dependent thermostability changes. Two of the residues (E236 and D237) reside in SW-III of Gαi while the third residue (E245) lies in the neighboring α3 helix. Mutation of E236 and D237 in SW-III promotes Gβγ release in HEK293 cells, supporting the role of these residues in pH-dependent electrostatic interactions that allosterically regulate heterotrimer formation. While both Ras and heterotrimeric G-proteins contain SWI and SWII regions, Ras proteins lack the SW-III region found in Gα proteins. The presence of this unique SW-III region in heterotrimeric G-proteins may explain the unique pH sensing properties of Gαi and provide a novel role for the SW-III region in pH-dependent Gβγ release from the Gαi-Gβγ complex. Of note, the pH sensing network identified in this study is distinct from the network predicted by Isom et al^3^. This computationally predicted pH sensing network consists of residues at the Ras/helical domain interface (e.g. K46, D150, D200, D229, R242, K270, and K277). However, our NMR and CD data do not support this interaction network, as observable NMR resonances associated with K270 and K277 do not show pH-dependent perturbations and mutation of K46 and R242 did not alter pH-dependent thermostability changes measured by CD.

Large changes in the stability of GTP-bound Gαi have previously been attributed to the formation of an interaction triad (termed as G-R-E Triad) involving residues G203 and R208 from SW-II and E245 from α3. This interaction triad is absent from the less stable and more dynamic GDP-bound state^7,25^. Interestingly, E245 is also one of the residues identified in the pH sensing network. Moreover, E236 and D237 from SW-III interact with SW-II via residue R205 to stabilize both Switches in the GTP-bound forms of the protein. This nucleotide-dependent disorder-to-order transition is predicted to facilitate the release of Gβγ from Gαi resulting in the activation of Gαi. Consistent with these findings, SW-III residues E236 and D237 are involved in pH-dependent electrostatic interactions that appear to allosterically regulate Gβγ release.

While several pH-sensing proteins contain titratable histidines^36,37,38^, others such as EmrE contain aspartate and glutamate residues that titrate in the physiological range due to the formation of electrostatic networks^32–39^. In these systems, even a small reduction in pH can alter the side chain protonation state leading to the neutralization of charge. In the case of Gαi, we identified a network of three charged residues (E236, D237 and E245) that regulate pH-dependent thermostability changes in the Gαi-GDP. To investigate how protonation/deprotonation of these residues might alter Gαi structure and dynamics in the GDP-bound state, we performed MD simulations. As starting points for the simulations, residues E236, D237 and E245 were generated in both charged and uncharged states, to represent higher (deprotonated) and lower pH (protonated) states, respectively. Results from these analyses suggest that the protonation of residues E236, D237 and E245 enhances Gαi-GDP dynamics due to the loss of electrostatic interactions between SW-II, SW-III, and α3. Moreover, we find that these residues make transient electrostatic interactions in Gαi-GDP at higher (~>pH 7.2), but not at lower pH. Since the binding of Gβγ to the Gαi depends on the dynamic properties of SW-II, we propose that lower physiological pH promotes Gβγ binding to Gαi resulting in attenuation of both Gαi and Gβγ mediated signaling pathways. Consistent with this premise, our findings indicate that higher intracellular pH_i_, promotes agonist-mediated Gβγ release from Gαi via the formation of pH-dependent electrostatic networks involving key residues within SW-III and α3. This in turn, will regulate Gαi and Gβγ downstream signaling.

While the current investigation is centered on the Gαi isoform, sequence and structural alignment with other Gα isoforms (such as Gαs, Gαo, and Gαq) reveals conservation within the core pH sensing network (Supplementary Fig. S6)^3^. This analysis suggests the potential for pH-sensing properties in other Gα isoforms. The development of pH-insensitive forms of Gαi will facilitate investigations of hormone- and neurotransmitter signaling during physiological stresses, such as occur during glucose or oxygen deprivation, leading to changes in cellular pH^40^.
